# Material Behavior and Fatigue Assessment of Old Steel Bridges of the Spanish Conventional Rail Network

**DOI:** 10.3390/ma14185275

**Published:** 2021-09-14

**Authors:** Álvaro Presno Vélez, Antonio Bernardo Sánchez, Octavio Ariñez Bruna, Diego Madera Abella, Laura Álvarez de Prado, Marta Menéndez Fernández

**Affiliations:** 1 Department of Mathematics, University of Oviedo, 33007 Oviedo, Spain; a.presno.velez@gmail.com; 2 Department of Mining Technology, Topography and Structures, University of León, 24071 León, Spain; laura.alvarez@unileon.es (L.Á.d.P.); marta.menendez@unileon.es (M.M.F.); 3 SERS Consultores en Ingeniería y Arquitectura S.A., 50008 Zaragoza, Spain; octavio@sers.es; 4 Laboratory Responsible, TAM Group, 33405 Avilés, Spain; diegomadera@eoti.es

**Keywords:** structural integrity, steel bridges, metallic structures, fatigue assessment, rail network, life expectancy of materials, fatigue life prediction

## Abstract

This work presented salient features of the steel behavior of seven metallic bridges close to, or over, 100 years old, among the Spanish conventional rail network as well as the results of a fatigue life expectancy study. A preliminary study of the properties of the constituent materials obtained from the bridges samples was carried out followed by dynamic fatigue tests under service representative loads. Due to the steelmaking techniques in the late 19th and early 20th centuries, disperse fatigue behavior results were obtained. However, the wide safety margins with which these bridges were designed, as well as the mechanical properties of the steel (relatively good mechanical resistance but with low ductility), seem to guarantee a long fatigue life. This estimate decreases sharply with increasing loads.

## 1. Introduction

For many decades, one of the most successful engineering solutions for railway networks has been the construction of metallic bridges. However, in parallel with a steep increase of traffic density on railway networks, some structural problems have begun to arise. One of the most concerning problems for these structures is that many of them are reaching or have exceeded their original design life. This fact, altogether with the growth of the average weights transported and the high number of stress cycles, has created the necessity of metallic railway bridges structural assessment (with special consideration to fatigue-induced damage).

With this intention, the bridge headquarters area of ADIF (Administrador De Infraestructuras Ferroviarias), a state agency managing railway infraestructure, in collaboration with the joint venture between “Tecnología y Análisis de Materiales S.L.” and “SERS, Consultores en Ingeniería y Arquitectura S.A.” began, in 2016, an extensive study, the acquisition of samples, and mechanical (static and dynamic) tests.

This work presented salient features of the material behavior of seven metallic bridges among the Spanish conventional rail network as well as the results of the fatigue life expectancy study. Fatigue has become one of the major issues associated with old metallic bridges and it can be estimated that it is responsible for 80% to 90% of failures in steel structures [1,2]. Fisher et al. [3,4] concluded that most fatigue cracks are caused by local vibration, distortion of member cross-sections, and out-of-plane bending of webs.

The fatigue life and structural material properties are among the most relevant engineering parameters for the determination of load-bearing capacity during operation. Other properties such as stress intensity, cyclic stresses, frequency, or manufacturing processes are usually studied to model and predict structural failure.

Fatigue assessment remains an open and relevant problem in many situations. The fatigue model is known as S-N curves or Wöhler’s curves (based on the Basquin equation [5,6] and considered in standards and codes aiming to characterize fatigue behavior both in high-cycle fatigue (HCF) and low-cycle fatigue (LCF) regions.

The model cleverly merges the influence of factors involved in fatigue life prediction, like the number of stress cycles or the stress level, and can be easily related to fracture mechanics.

If knowledge of material properties is essential while predicting the remaining fatigue life, this need is even more important in the case of metal structures that have been around for several decades. More precise measurements and analysis are required to estimate the behavior of these old materials facing dynamic loads. In that line, recent works on Portuguese metallic bridges [7,8], propose probabilistic approaches to deal with material models uncertainties.

Typically, fatigue data are studied in regions of 10^3^ to 10^6^ cycles. However, LCF or HCF regions can be prioritized. Most roadway or rail bridges, offshore structures, huge civil engineering constructions, or logistic structures are designed in HCF regimes [9]. Despite some recent studies [10] suggesting the use of S-N or ε-N curves also covering the LCF regime (i.e., earthquakes), the present work only considered applicable the HCF regime [9]. Therefore, estimations of the fatigue damage parameters such as Smith-Watson-Topper or Walker-Like were not included [10].

In most western countries, the assessment of existing rail bridges is based on existing codes and guidelines meant for the design of new bridges [11]. This leads to conservatism, which encourages the classification of these structures as deficient, creating great economic impact [11]. Some guidelines have been published that deal with the assessment of existing bridges in Europe: European Cooperation in Science and Technology (COST) 345 (COST 2004); European Commission 4th Framework Programme (EU FP5 2005 [12]) or EU FP6 SB-LRA [13] (EU FP6 2007b [14]). Unfortunately, all of these guidelines are meant for not-so-old highway bridges and do not consider the specific aspects characteristic of old railway metallic bridges, including materials and constructive details (such as riveting joints). The literature on the matter, including studies on bridge parts made of old puddled iron (or wrought iron) [15,16,17,18] and old assembly elements (such as rivets) [19,20], was examined.

The use of the riveting technique is discontinued for modern metal constructions; due to this, the knowledge concerning riveted structures’ ability to withstand fatigue efforts has not been so deeply researched. According to Cremona (2013) [11] and Wichtowski (2015) [21], fatigue results on girders or stringers in bridges showed to be (if severely corroded specimens are removed) over the Eurocode No.3, part 1.9 (CEN 1993c), detail category C = 71 N/mm^2^. For truss girders, results are better estimated with detail category C = 63 N/mm^2^ (EU FP6 SB4.6 (EU FP6 2007c) [22]).

Early metal bridges, until the end of the nineteenth century, were fabricated mostly of puddle or cast iron. Early mild steels succeeded them around 1895. EU FP6 SB4.6 (EU FP6 2007c [22]) go deep in terms of chemical composition, microstructure, and mechanical properties, highlighting a large amount of slags inclusions and anisotropy in the manufacturing process.

Alternative fracture mechanic methods can be used for the determination of operating time intervals for the prevention of unforeseen fractures within the inspection scheme. If two magnitudes are determined, a_o_ and a_crit_, the first being the smallest crack detectable by the available inspection methods and a_crit_ the critical length of the crack that triggers failure, it is possible to estimate the maximum permissible number of load cycles for a steel member under a certain fatigue load, giving a hint of the residual service life. Analogously, the accumulated damage attributable to past traffic is satisfied by the assumption of a crack of a defined crack size [23].

Fatigue assessment and fracture mechanics were used in the estimation of the remaining service life after material properties characterization for the metallic bridges.

### 1.1. Description and Background: The Bridges of the Rail Network under Study

**Figure 1:**
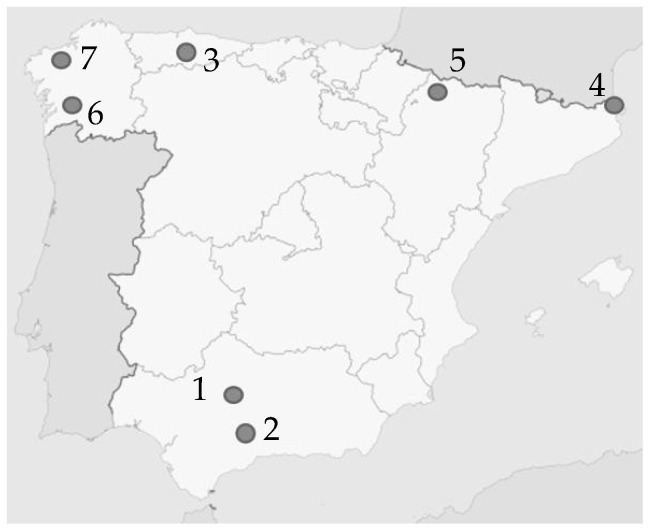
Approximate location of the bridges. 1.”Bembézar” PK 41/548 “Cordoba-Sevilla” line; 2. “Corbones” PK 528/318 “Madrid-Sevilla” line; 3. “Caudal” PK 018/525 “Trubia-Collanzo” line; 4.”Colera” PK 270/500 “Tarragona-Barcelona” line; 5. “La Peña” PK53/161 “Huesca-Jaca” line; 6. “Carballo” PK 0/909 “Redondela-Santiago de Compostela” line; 7. “Tea” PK 129/493 “Chapela-Lemos”.

There are currently 201 riveted metal bridges managed by ADIF with a span of more than 20 m on the Spanish railway network (those identified as relevant to the nature of the present work). Covered under the scheduled maintenance, samples of seven metal bridges (Figure 1) were obtained that contain examples of the various materials and manufacturing codes used since the late 19th and early 20th centuries (1884–1927).

The present study was carried out through tests on materials extracted from seven bridges. The authors consider that the description and background of the seven bridges would be too long. That is why two bridges were chosen based on their representativeness (“Corbones” and “Bembézar”). An analogous previous study was carried out on the other five bridges.

### 1.2. Description: Bridge “Arroyo Corbones” (Corbones’ River)

The “Arroyo Corbones” metallic bridge is located at PK 528/318 of the Madrid-Seville railway line, in a section with a double-track platform. It consists of two independent metallic sections, one on each road.

**Figure 2:**
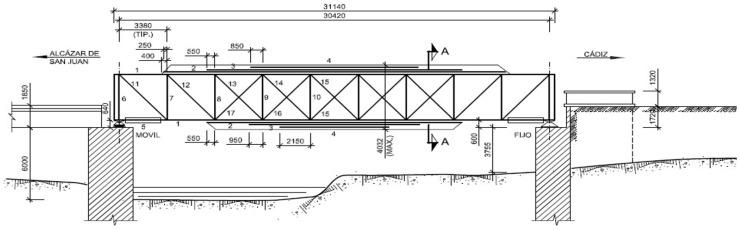
“Arroyo Corbones” elevation sketch (dimensions in mm).

The metal section that was the object of study corresponds to that of the left road, which is the one that receives the passage of exceptional transports and therefore a higher mechanical load. It is a single-span bridge with 30.42 m between support axes and 31.14 m of beam length, in the Pratt beam typology with nine squares with the five central squares arranged contradiagonal, according to the arrangement reflected in the following sketch (Figure 2).

The maximum depth of the structure is 4.03 m, approximately 3.6 m between the centers of gravity of the main chords, which translates into slenderness of approximately 1/8, typical of bridges built under “high load requirements”, one of the categories of the Instruction of 1925 [24]. The number of squares marks the separation of the joists, 3.38 m, very similar to the edge, which means that the diagonals of the lattice are approximately 45°. The main beams consist of upper and lower chords with a T-arrangement and addition of horizontal blades towards the light center areas, with a single 500 mm × 12 mm web and horizontal plates 400 mm wide and 10 or 11 mm thick.

**Figure 3:**
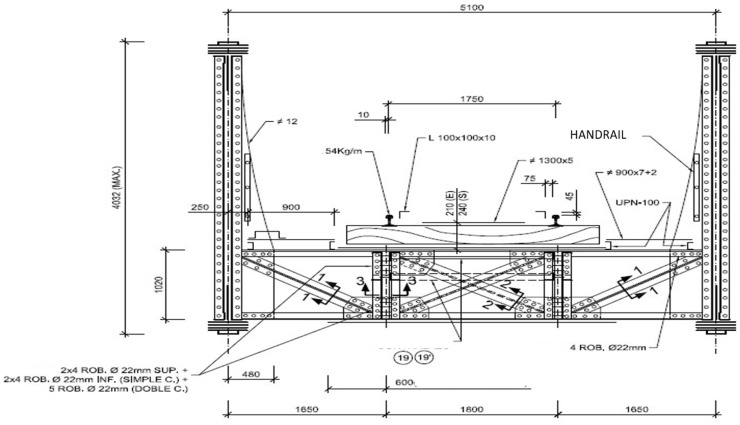
“Arroyo Corbones” transversal section sketch (dimensions in mm).

This original arrangement of the main chords has been modified by the successive addition of other pieces arranged to reinforce the original section. The uprights and diagonals are formed by the juxtaposition of angles, with more powerful sections as they approach the supports. The main beams have backlash diagonals in the five central squares, made up of the juxtaposition of two angles (Figure 3).

For the dating of the bridge, it was possible to find the original documentation of the current metallic sections in the archives of the National Railway Museum. The carrying out of the load test for this metal section was on 25 January 1921, for the right track. The left one was included two decades later (samples were obtained from this last one).

### 1.3. Description: Bridge “Bembézar”

The “Bembézar” metallic bridge is located at PK 41.548 of the Córdoba-Seville railway line, in a section with a single-track platform. It is a bridge with two independent oblique spans with a span of 23.40 m, a beam length of 24 m, and a 20-m span without skew slightly separated from the other two.

**Figure 4:**
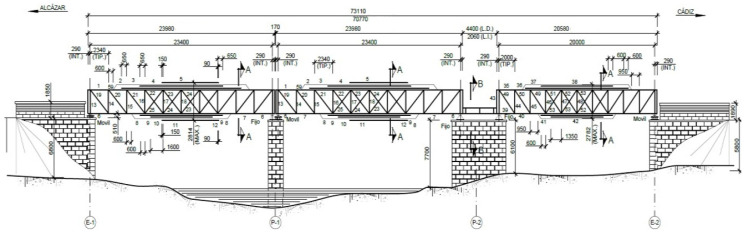
“Bembézar” elevation sketch (Dimensions in mm).

This bridge has the peculiarity that the distance between the end of the second span and the beginning of the third, in the abutment area is also saved by spars of different lengths given the skew of span in section two and the absence of skew in the span in section three, thereby achieving continuity of the metallic section (Figure 4).

The typology of the metal sections is a Pratt beam with a lower deck (passing the train between the main beams). In the metal sections of 24 m in beam length (23.40 m between support axes), the maximum depth of the structure is 2.814 m, and in the 20 m. of light, the depth is 2.782 m (in reality it is 2.35 m between the centers of gravity of main heads).

**Figure 5:**
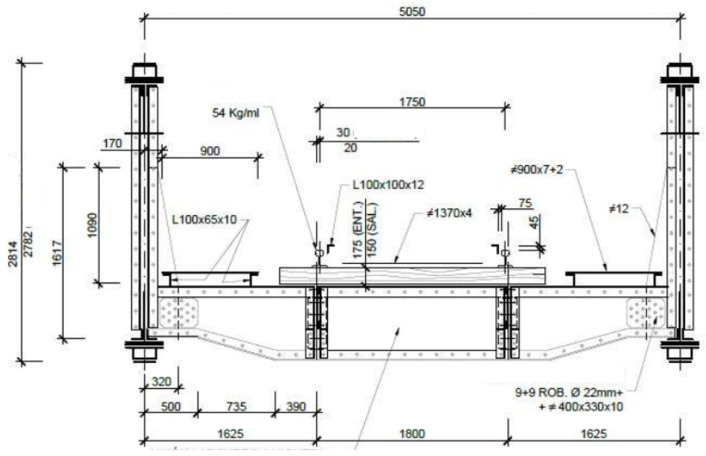
“Bembézar” transversal sketch (Dimensions in mm).

In all of the cases, the metal sections consist of 10 squares, which marks the separation between joists, which is at a tenth of the span, that is, 2.34 m and 2.00 m (Figure 5).

The uprights and diagonals are formed by the juxtaposition of angles, with more powerful sections as they approach the supports.

The bridge is heir to the primitive work executed prior to the episode of the “Bembézar” river flood during the last eight days of December 1887 (of which there is evidence) and the “final” configuration dates from 13 November 1918.

### 1.4. Initial Calculation

The calculations for the “Arroyo Corbones” metal section of the left track are dated from 2 November 1927, and are in accordance with the criteria and ways of calculation as per the “Instruction for the drafting of projects of metal sections” of 1925 [24].

In the case of the “Bembézar” bridge, the calculations of the metallic sections are dated on 18 January 1917, therefore adjusted to what is indicated in the, even older, 1902 instruction [25]. This instruction was in place at that time and estimated margins of around 20% in the main beams.

In any case, the results showed moderate working stresses obtained from load postulates which are much higher than the current requirements, which would indicate that the sections of these bridges should not have problems withstanding current loads, working at low tension levels.

This is an expected result considering the over-calculation margins used at the beginning of the century.

### 1.5. Fatigue Live Estimation

ADIF’s rail network technicians, based on traffic records on the “Lora del Río-Sevilla” line, had suggested the date presented in Table 1 to estimate the accumulated load cycles on the different bridge’s elements. Traffic frequency has been magnified by 50% to account for significantly higher rail traffic density in earlier times (historical records were not complete). For fatigue purposes, a differentiation was made between elements of the main chord, for which each load cycle is estimated by the number of trains that cross the track, and the elements of the load floor, whose cycles are related to the total number of axles (stringers) and bogies (joists).

An average composition was made for freight trains being 6-axle locomotive and 14 wagons with 28 bogies or 56 axles, for a total of 30 bogies and 62 axles, while passenger trains being a 6-axle locomotive and five cars with two bogies and four axles (to bring the estimate closer to the average that has been available since 1917), which leads to 12 bogies and 26 axles. Approximately 65% of traffic are passenger trains while 35% are freight. Table 1 presents an estimation of the number of cycles for 90 years of operation.

## 2. Materials and Methods

### 2.1. Sampling

The following material was extracted from each of the bridges to characterize the properties of the materials: four specimens of 400 mm × 100 mm from the main chord plate and seven to eight samples of 100 mm × 100 mm × 10 mm from an 850–900 mm L-beam.

**Figure 6:**
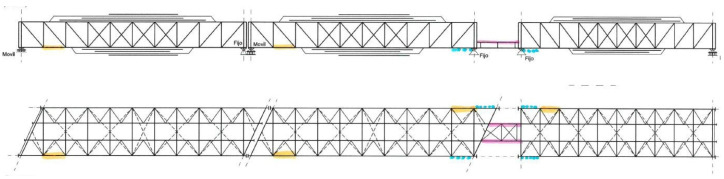
“Bembézar” sampling sketch. Yellow: bottom chord of the main beam, Blue: bottom chord of external beams on the 2nd pile, Pink: stringers on the 2nd pile.

**Figure 7:**
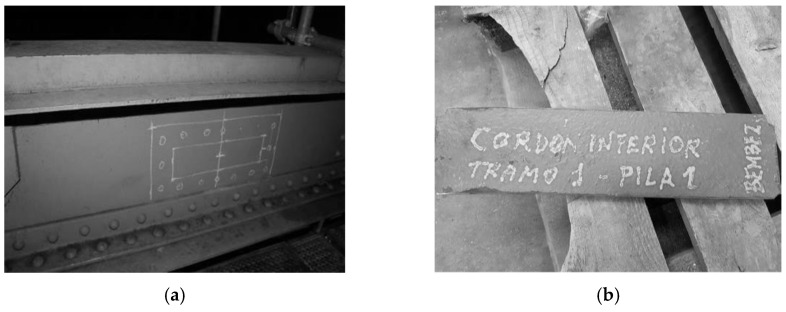
(**a**) Sample position example from the “Bembézar” bridge; (**b**) Sample example after extraction from the “Bembézar” bridge.

Efforts were made to extract materials from both the main elements and the load floor but always while having into account the current situation of the bridges and how to minimize operations depending on the location. Figure 6 sketches the extraction zones for the sampling. Figure 7 shows some sample examples after extraction.

### 2.2. Experimental Design and Testing

This article contains a study of the fatigue behavior of the constituent material of old metal railway bridges, as a preliminary step to determine their residual life.

The study carried out was structured in four phases as it is presented in Table 2. In the first, a preliminary study of the properties of the constituent materials obtained from the bridge samples was carried out.

**Mechanical resistance:** Mechanical resistance plays a fundamental role in and forms a constitutive part of fracture mechanics [26]. Additionally, the determination and control of its value is a fundamental part of the quality control of the material properties (for structural materials). Tensile tests were performed on all samples according to European standards on all-size specimens.

**Resilience:** Charpy simple-beam V-notch impact tests were also performed on the samples. CVN specimens 10 mm × 10 mm were used. All tests were performed at 0 °C with a 300 J pendulum device.

**Chemical analysis:** The chemical composition of the material is a well-known factor that exerts influence on mechanical properties. Samples were analyzed by optical emission spectrometry (Spectromax metal analyzer). Results were statistically processed to offer the best-weighted average estimator considering the different uncertainties of the testing method and for the following elements: C, Mn, Si, P, and S.

**Microstructure:** Some authors [27,28] have studied the relation between microstructure characteristics and fracture mechanics properties and supports, and the influence of grain size, angle of grain boundaries, orientation, and inclusions on the nucleation and propagation of cracks.

In the second phase, the parameter “m” from the Paris’ Law was estimated with pre-cracked (one-side) SE(B) specimens tested with a clip-on extensometer IB-3541-008M-040M-ST-E339 (Madrid, Spain) and software according to ASTM E647 [29]. The crack growth was measured by optical microscopy. The aim was to assess the influence of the defects present in the samples, both metallurgically and related to their mechanical behavior. Additionally, the results were compared with a similar study carried out in 1992 [30] with positive results.

In the third phase, the fatigue behavior was taken as an objective. To try to reproduce the work of the metal parts on the bridge as faithfully as possible, it was decided to implement a test in which the specimens of the material were subjected to pure single-axle traction. The tests were performed on a flat-sheet fatigue specimen with a rectangular cross-section as per ASTM E606 (a) with thickness as received (8–10 mm). It was found that the specimens did not experience fatigue failure, despite taking the test to a high number of cycles (10 million), either by the absence of nucleation of a crack or by the low or no advance of this.

Therefore, based on the real case reproduction approach, it was finally decided to carry out a double-notched pure tensile test, thus triggering the appearance of a crack and representing potential stress-intensity factors on the real bridges (2 mm deep v-notch on each side with 0.025 mm radio on the tip on a whole section of 10 mm × 8 mm).

This beginning of cracking can respond to the real situation that exists in certain elements of the bridge in which, due to lamination defects of the pieces, dents, scratches, or as a result of drilling, repairs, or any other cause, there is a specific defect that triggers the crack. The current maintenance non-destructive surface testing of the bridges should allow fully formed cracks to be detected (and monitored or repaired) up to a reasonable detection limit.

Regarding the stress range used in the tests and in order to match, as far as possible, with the real working situation on the bridge, it was decided to consider load steps of 100 MPa, 150 MPa, and 200 MPa, starting with the highest stress first and following the suggestions of the historical design codes.

If from the tests carried out it was deduced that the material did not collapse, no additional tests were carried out for lower load steps.

However, it is pointed out that the objective of the present study was to find the number of failure cycles based on empirical testing and not according to a theoretical model based on the Paris Law or any other method with assimilable theoretical curves.

## 3. Results

### 3.1. Preliminary Properties Characterization

Mechanical initial characterization of the material properties is shown in Table 3.

Regarding the yield point, the average R_p 0.2_ value obtained for “modern” steel was 267.2 MPa, for steel between (1917–1920) 296.5 MPa, steel from (1905) 306.2 MPa, and older puddle iron 312.8 MPa. All of them showed mechanical properties much higher than the minimum required at the time of its construction. Furthermore, the variability of the results was very low, which statistically is also favorable for characterization.

In the main chords, some values obtained are somewhat lower than the stringers, for example, in the case of the “Bembezar bridge”.

The UTS (Ultimate Tension Strength) in beams, with an average value of 382.3 MPa and a minimum of 332 MPa, is much closer to the requirements that were in place at the time of the bridge’s construction.

In addition, the result is well above what is required in the regulations of the time in terms of elongation at break, with constant values of 33–34%, which leads to a quality coefficient of around 13.

The EC1990 standard also establishes that approximate design periods of use for bridges (Category 5) are set to 100 years. Steel structures should have sufficient toughness to withstand various operating temperatures. Standards relating to steel products generally require that the impact energy of the Charpy V-notch test, at a given test temperature, is not lower than 27 J (according to PN-EN 10025 [31]). With thicknesses of up to 30 mm, Eurocode 3 requires that the temperature is −20 °C for steel bridges. However, this requirement applies to new welded structures designed from modern structural steels with high-impact parameters, taking into account the material’s resistance to brittle fracture throughout the expected service life. Hence, the question arises of how we should treat structures that have reached the age of over 50 or even 100 years [32].

Regarding the resilience test, tests have been carried out at 0º since it is understood that it can be associated with a feasible working temperature at the point of location of the bridges. The poor result obtained in this test was confirmed, with mean values around 19 J for the stringers and even lower, around 11 J, for the main chord samples, whereas according to current regulations twice as much would be required (27 J).

Chemical analysis results were approximately in the range of S235 steel (carbon content 0.2% compared with the maximum allowed 0.19%), while the rest of the content of parameters were within the values indicated in the table of the standard, with special mention regarding the sulfur content, quite variable, with a minimum of 0.018% and a maximum of 0.12% against a usually permitted 0.045% or 0.035% (for modern construction steel).

According to the tests obtained, the constituent material of the bridge is more similar to the steels from the beginning of the century, with low carbon content and notable presence of impurities, than those from the years prior to the Civil War (period 1925–1936) that are already much closer to the modern ones.

### 3.2. Microstructure

**Figure 8:**
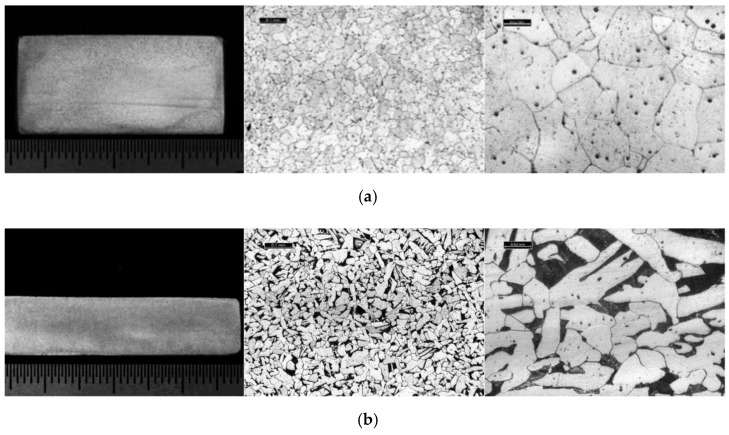
Macrographic and micrographic results for both studies. (**a**) From left to right: Macrographic, micrographic 100×, micrographic 500× “Bembézar” Stringer sample; (**b**) From left to right: Macrographic, micrographic 100×, micrographic 500× “Corbones” Stringer sample.

The microstructure is mainly formed by grains of ferrite, a characteristic structure of mild steel with very low carbon content. Small areas of the pearlitic matrix and traces of tertiary cementite can be seen in the grain joints. Again, a considerable number of non-metallic inclusions appear derived from the chemical reaction of the impurities present in the chemical composition of the material, such as manganese sulphides, oxides or silicates. No macro segregations or macroscopic defects such as sheet separation or porosity were observed. Figure 8 shows some representative examples of macrographic and micrographic (100–500×) examination.

### 3.3. Fatigue

**Figure 9:**
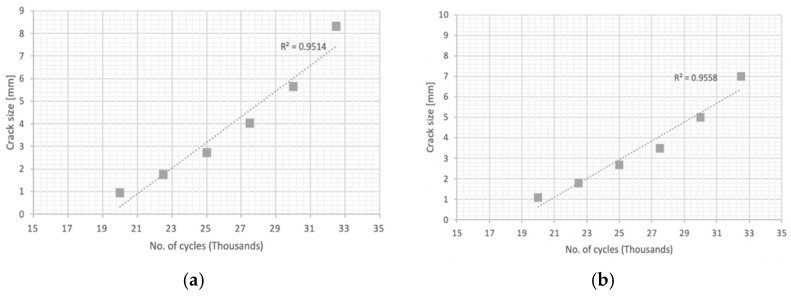
No. of cycles: crack size (**a**) Corbones Stringers; (**b**) Corbones Main Chords.

**Figure 10:**
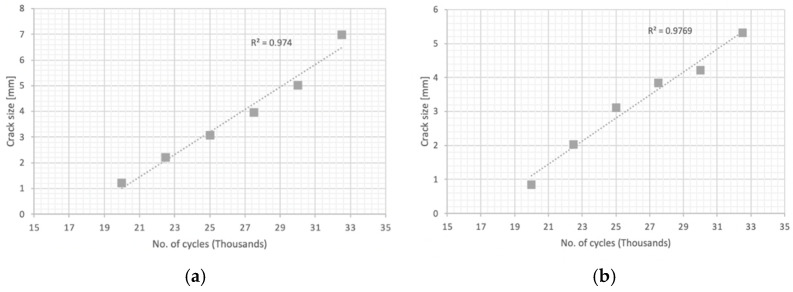
No. of cycles: crack size. (**a**) Bembézar Stringers; (**b**) Bembézar Main Chords.

**Figure 11:**
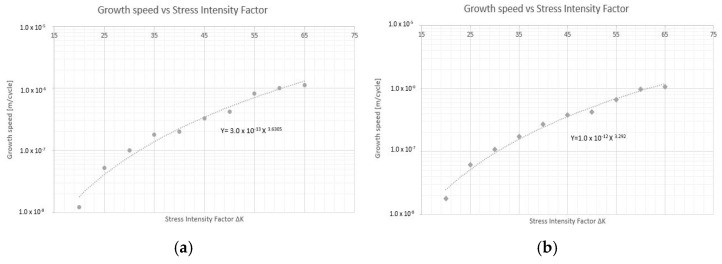
Growth speed vs. intensity factor. (**a**) Corbones Stringers, m = 3.63; C = 3 × 10^−13^; (**b**) Corbones Main Chords, m = 3.29; C = 1 × 10^−12^.

**Figure 12:**
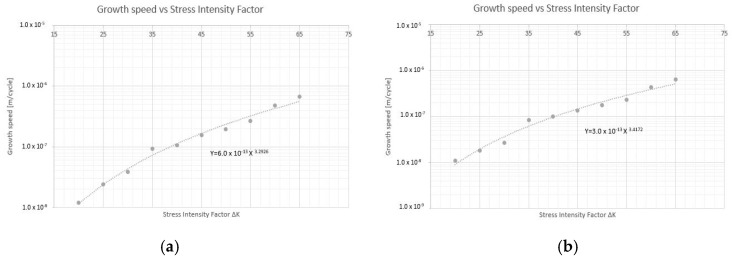
Growth speed vs. intensity factor. (**a**) Bembézar Stringers, m = 3.29; C = 6 × 10^−13^. (**b**) Bembézar Main Chords, m = 3.41; C = 3 × 10^13^.

**Figure 13:**
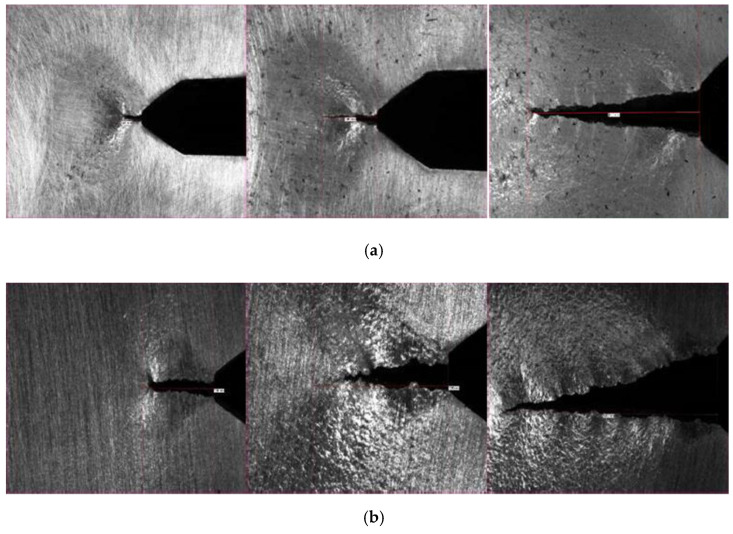
Crack growth sequence example (**a**) Bembézar Stringers; (**b**) Bembézar Main Chords. The width of each image corresponds to 5 mm.

In the first place, fracture mechanics tests were carried out to determine the constant “m” that governs the speed of propagation of the crack according to the Paris’ law, by means of a test on a pre-cracked sample supported at two points with a center load. Figure 9 and Figure 10 plot the no. of cycles vs. the crack size for pre-cracked SE(B) samples. Figure 11 and Figure 12 show the relation between crack growth speed and the intensity factor. It is important to highlight that phase two was carried out only on samples from the chords and stringers of “Bembézar” and “Corbones”. Figure 13 shows crack growth sequence examples.

For phase three, fatigue tests equivalent to 100 and 200 MPa were carried out using a flat-sheet fatigue specimen with a rectangular cross-section. In all cases, for the loads used, no crack was generated after 10^7^ cycles, so it was estimated to be below the natural endurance limit of the material. Therefore, it was insensitive to fatigue failure unless it has some initial imperfection that triggered the appearance of the crack, after which the crack spreads.

**Figure 14:**
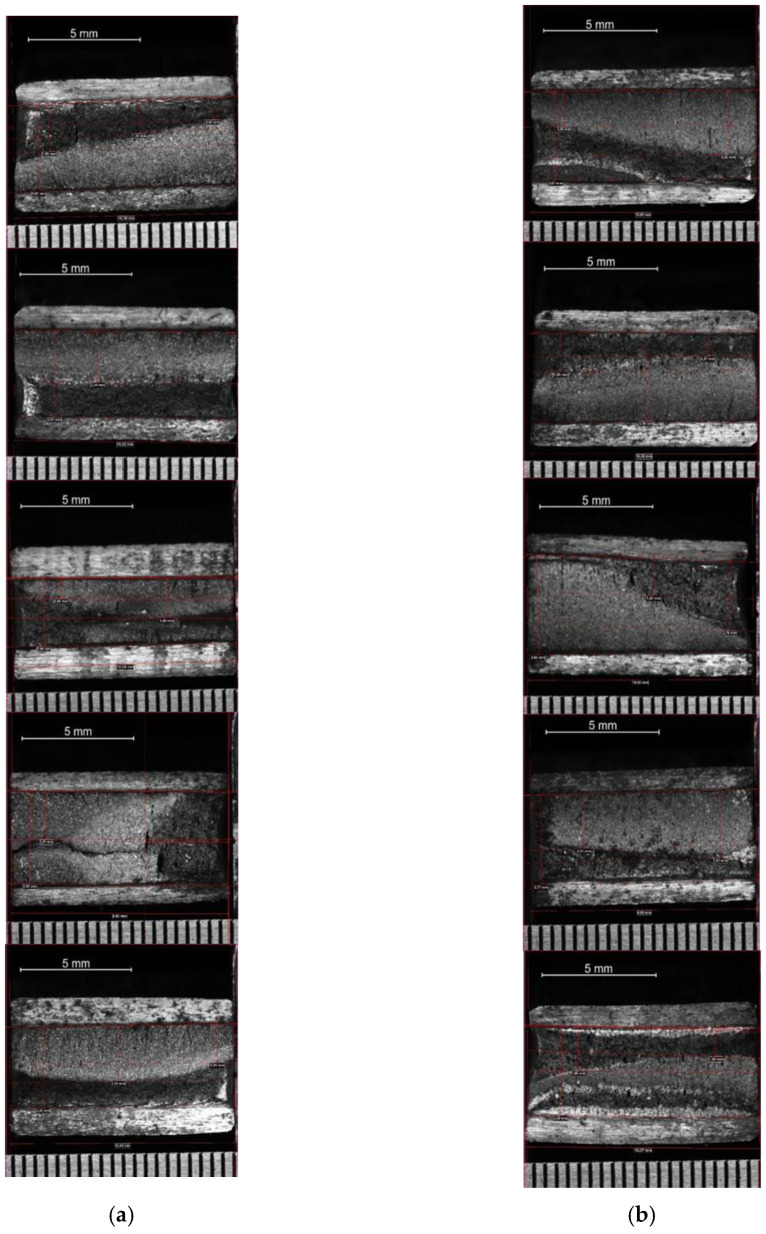
Sample fractography (**a**) Corbones Stringers; (**b**) Corbones Main Chords.

**Figure 15:**
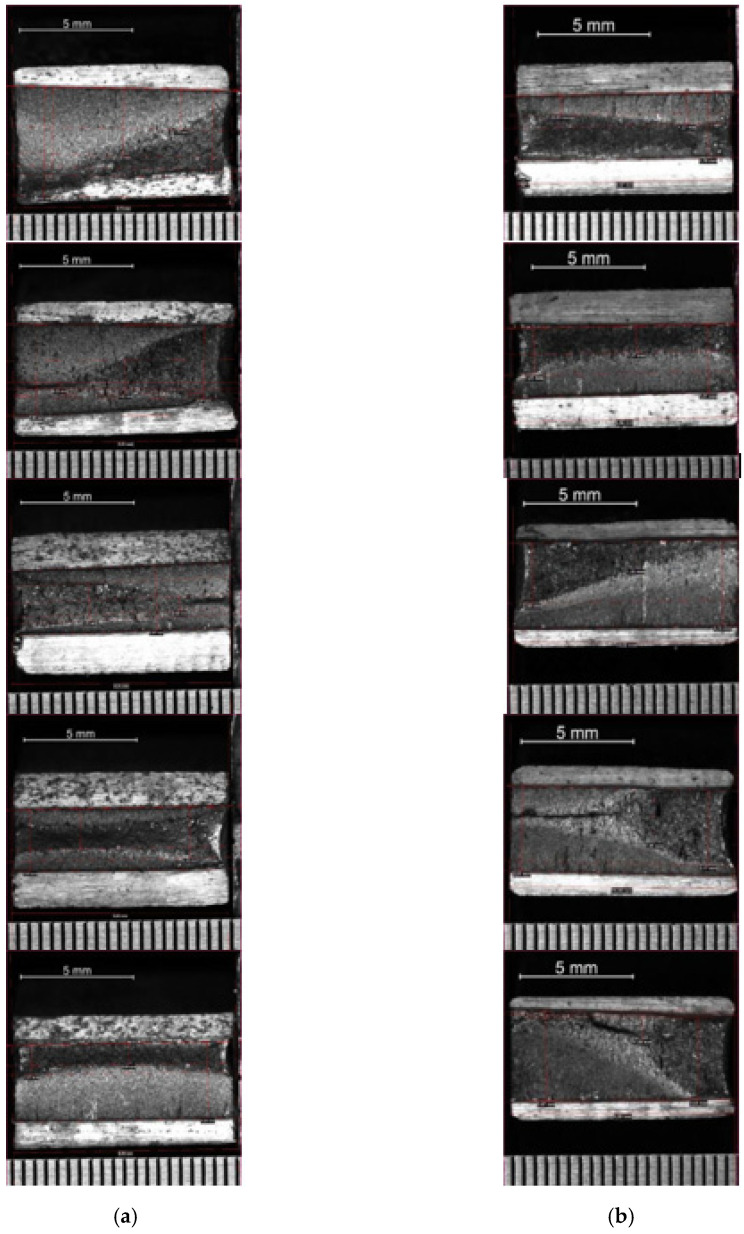
Sample fractography. (**a**) Bembézar Stringers; (**b**) Bembézar Main Chords.

The studies continued with the fourth phase by incorporating a double notch in the specimens. Doing so, the section continued to be symmetrical, and no bending appeared that could make the interpretation of results more complex but, at the same time, the notch triggered the appearance of a growing crack. On the following tables and figures (Table 4, Table 5, Table 6 and Table 7 and Figure 14 and Figure 15) the results for fatigue tests on double-notched samples are shown along with fractographic images after the break. This test was performed on samples from the seven bridges. The results for the “Bembézar” and “Corbones” bridges are presented explicitly. The other results are summarized in Table 8.

**Figure 16:**
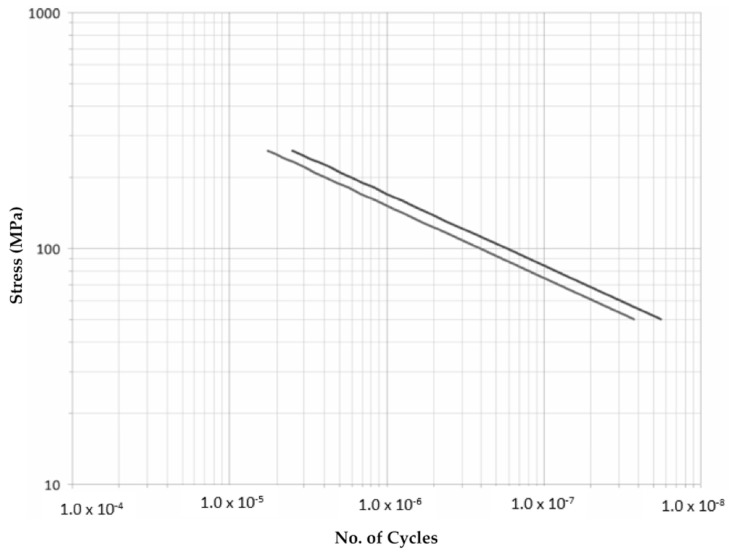
Corbones S (MPa)-N approximation. Dark: Strings, Light: Chords.

**Figure 17:**
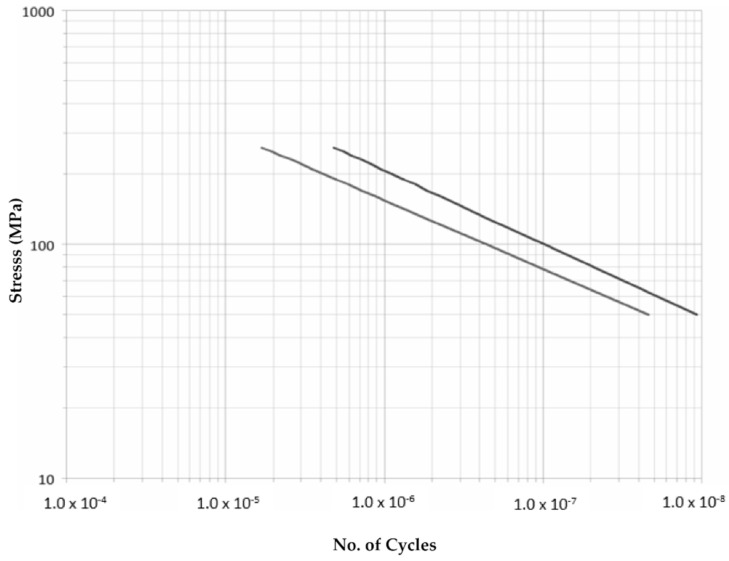
Bembézar S (MPa)-N approximation. Dark: Strings, Light: Chords.

The results obtained allow an extrapolation regarding the number of cycles reissued at tensions other than 200 MPa according to the theoretical behavior collected by Paris’ law. We opted for such a simple model due to its ubiquity and easy correlation. The results are displayed in Figure 16 and Figure 17.

## 4. Discussion

### Whole Sample Selection Results

The main data obtained in the tests carried out on the materials extracted from the seven metal bridges are presented in Table 8. The results offered are expressed as arithmetic means. In relevant cases, the range of results is expressed between brackets. The fatigue resistance of the material is expressed as required fatigue cycles to induce a crack growth of 1 mm. Again, this measurement is an average value.

Table 6 above shows aspects that are consistent with those that could be deduced from the results of the tests carried out at the end of the 1980s [30] to characterize the material of metal bridges in Spain.

The steel used in metal bridges after 1925 is characterized by higher carbon content, generally not less than 0.2%, and a lower content of impurities (phosphorus and sulfur among other elements). The evolution of the processes made it possible to standardize results but also to partially improve safety margins in terms of resistance parameters so that steels from the “intermediate” period (“La Peña”, “Colera”) offer higher elastic limits and even significantly higher ultimate tensile strength (UTS).

The results in terms of ultimate tensile strength were in most cases insufficient with respect to the current requirements, while in all cases the elastic limit was above the values of the respective regulations in place when they were design. The “puddle iron” material offers a mechanical resistance comparable to some modern steel; however, it is betrayed by its lower ductility and the propensity to present lamination defects.

The results of the fatigue tests did allow us to obtain clear correlations between the fatigue behavior of the components and when they were manufactured. In fact, the results obtained in the “Río Tea” and “Corbones” bridges (some of the newest) were in the low range of resistance on fatigue tests with an amplitude of 200 MPa.

There seems to be a certain relationship between the values found for the elastic limit and the results compared to the fatigue test, in the sense that elements that offered higher elastic limits offered good performance, while parts with lower elastic limits offered more disperse results.

**Figure 18:**
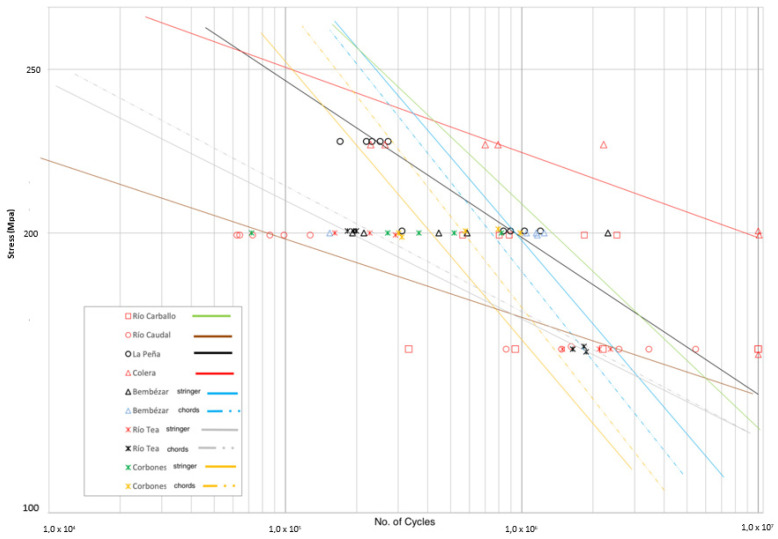
Summary of results. Stress (MPa) vs. No. of cycles for double-notched uniaxial fatigue tests (frequency: 10 Hz, R: 0.1).

Finally, the evolution of the number of cycles resisted for the amplitude of loads is shown in Figure 18, where all the values obtained in the tests are shown together. It should be noted that in the case of the “Corbones” and “Bembézar” bridges, the values given by the Paris’ law were represented based on the results obtained on a 200 MPa amplitude tension test, since no tests were performed on other loads.

The slope of the lines reflects an aspect of great relevance, such as the influence that a variation in the applied load has on the number of cycles resisted.

In this sense, the results obtained in all cases offered more stretched lines than those represented for the Corbones and Bembézar bridges given by Paris’ Law, for which values of the k coefficient between 3.19 and 3.4 were found. The results obtained in other bridges led to significantly higher values of the k coefficient, 4.57 in the metallic material of the “La Peña” bridge but 7.7 in the main chord material in “Río Tea” and up to 8.6 in the “Colera” bridge. However, the material of this bridge shows great behavior against fatigue (right and upper part of the graph).

On the contrary, for the “Río Caudal” bridge on the bottom-left side, an especially unfavorable behavior to fatigue is shown (theoretically not would withstand cycles at 250 MPa amplitude).

The graph above reflects that, at least in terms of the results found in the tests, it is not uncommon to find values of the coefficient “m” higher than the theoretical values determined by Paris’ law and reflected in the literature as normal values between three and five, which results in a more rapid loss of resistance to fatigue with the increase of load amplitude. This can be explained by the presence of impurities or defects in the material.

## 5. Conclusions

Several tests were carried out to characterize the metallic materials on seven bridges of the Spanish conventional rail system. The historical research carried out on the bridges has made it possible to distribute them among different categories based on the codes and standards used on their design and calculation and the constituent steel used in manufacturing.

- In this context, the materials used in the metal bridges were puddled iron until the end of the 19th century, and steel in its different qualities from the end of the 19th century (exclusive steel during the 20th century).
- The evolution in the material resulted in greater control of the manufacturing processes, which allows less dispersion of results, and a precise adjustment to the requirements imposed by the regulations in place (“Instructions for drafting metal bridges projects from 1902 and 1925”).
- It was found that, in some cases, there was a reduction in the mechanical properties of the manufactured material, and as the result of the product was controlled with greater precision (that is to say, more modern steel).
- In all samples without a notch, for the initial loads used, no crack was generated after 10^7^ cycles, so it was estimated to be below the natural endurance limit of the material. Therefore, it was insensitive to fatigue failure unless it has some initial imperfection that triggered the appearance of the crack, after which the crack spread. Under these conditions, new notched specimens were subjected to load cycles of amplitudes of 150 MPa, 200 MPa, and 250 MPa, depending on the elastic limit deduced in the corresponding tests and line with the load estimations obtained from the design structural calculations.
- In all cases, higher results were obtained when the amplitude of the stresses on the fatigue tests remained 100 MPa below the elastic. The next step, 50 MPa below the elastic limit, led to a significant decrease in the number of cycles until fracture (a reduction even higher than predicted with Paris’ law). These results highlight the importance of maintaining this margin of 100 MPa, a margin that was very present in the design codes of 1902 and 1925 where, with an elastic limit requirement of 220 MPa and 250 MPa respectively, the tensions in the pieces were limited to 100–110 MPa and 140 MPa.
- Puddled iron material, despite its low ductility, which is an intrinsic drawback of the material, did not present worse fatigue behavior: quite the contrary, although the tendency to present lamination defects that result in the appearance of relatively premature breaks should be noted.
- At least among the tested bridges, the best performance was observed in bridges from 1910–1920, with those after 1925 showing moderate or even poor performance, not only in terms of fatigue but also in terms of other mechanical parameters. In return, the low values obtained in these bridges presented a much lower dispersion.
- The presence of phosphorus and sulfur impurities did not result in a decrease in the performance of the fatigue parts.
- Finally, and concerning the propagation speed of the crack, conclusions similar to those of the number of cycles were observed, with a great dispersion of results.

## Figures and Tables

**Table 1:** Accumulated no. of load cycles on the bridges (expressed as no. of cycles).

**“Arroyo Corbones”**	**Estimated for 90 Years + 50%**
Elements of the main chords:	1.68 million cycles
Side members elements:	55.1 million cycles
Joist elements:	25.9 million cycles
**“Bembézar”**	**Estimated for 90 Years + 50%**
Elements of the main chords:	1.1 million cycles
Side members elements:	41.2 million cycles
Joist elements:	19.5 million cycles

**Table 2:** Experimental design and testing summary.

1st Phase	2nd Phase	3rd Phase	4th Phase
Preliminary Mechanical Tests	Fracture Mechanics	Fatigue (Un-notched)	Fatigue (Double-notched)
Macrographic and Micrographic examination according to ASTM E112 (2013). Preparation of the sample according to the guide E3-11. Chemical content of C, Si and S by optical emission spectrometry (SpectroMax Metal Analyzer) Tensile test according to UNE-EN ISO 6892-1: B (Average of three or two tests depending on the sample availability) Toughness measured by Charpy V-notch UNE-EN ISO 148-1 CVN specimens 10 × 10 mm. Temperature of 0 °C. 300 J Pendulum.	Determination of the “m” coefficient Paris Law expressed as: da/dN = C(ΔK)^m^ Pre-cracked (one-side) SE(B) specimens tested with a clip-on extensometer IB-3541-008M-040M-ST-E339 and software according to ASTM E647. Crack growth measured by optical microscopy. Machine: Dynamic tensile machine UFIB-200E-MD5W	Flat-sheet Fatigue Specimen with rectangular cross section as per ASTM E606 (a) Thickness as received (8–10 mm) Machine: Dynamic tensile machine UFIB-200E-MD5W Frequency: 10 Hz R: 0.1 No. of Cycles: 10^7^	Flat-sheet Fatigue Specimen with rectangular cross section as per ASTM E606 (a) Thickness as received (8–10 mm) Notch: 2 mm deep, 45 degrees and R0.25 mm Machine: Dynamic tensile machine UFIB-200E-MD5W Frequency: 10 Hz R: 0.1 No. of Cycles: To failure
Tests performed on the following metal bridges (seven): “Bembézar” (Stringers + Chords) “Corbones” (Stringers + Chords) “Caudal” (Stringers) “Carballo” (Stringers) “La Peña” (Stringers) “Colera” (Stringers) “Tea” (Stringers)	Tests performed on the following metal bridges (two): “Bémbezar” (Stringers + Chords) “Corbones” (Stringers + Chords)	Tests performed on the following metal bridges (seven): “Bembézar” (Stringers + Chords) “Corbones” (Stringers + Chords) “Caudal” (Stringers) “Carballo” (Stringers) “La Peña” (Stringers) “Colera” (Stringers) “Tea” (Stringers)	Tests performed on the following metal bridges (seven): “Bembézar” (Stringers + Chords) “Corbones” (Stringers + Chords) “Caudal” (Stringers) “Carballo” (Stringers) “La Peña” (Stringers) “Colera” (Stringers) “Tea” (Stringers)

**Table 3:** Mechanical initial characterization of the material properties.

	Puddle Iron	Steel (1905–1910)		Steel (1917–1920)			Steel (>1925)			
Metallic Bridge:	“Carballo”	“Caudal”	“La Peña”	“Colera”	“Bémbezar” Stringers	“Bémbezar” Chords	“Tea” Stringers	“Tea” Chords	“Corbones” Stringers	“Corbones” Chords
YS (0.2%) (MPa)	312.8 (297–324)	283.4 (272–289)	329.2 (321–342)	313.2 (271–351)	269.3 (261–277)	307 (300–314)	253.6 (242–266)	-	293 (289–299)	255 (254–356)
UTS (MPa)	367.4 (355–375)	381.2 (375–388)	418 (416–421)	379 (358–400)	332 (331–333)	367 (366–368)	405 (337–463)	-	409.3 (404–418)	382.5 (380–385)
Elongation (%)	9 (8–10)	35 (34–36)	32 (30–33)	35 (30–42)	39 (37–41)	32 (31–32)	30 (26–32)	-	34 (33–34)	35 (35)
C[w/w%]	0.016	0.068	0.095	0.049	0.029	0.054	0.0252	0.074	0.02	0.012
P[w/w%]	0.12	0.065	0.084	0.123	0.036	0.054	0.012	0.014	0.006	0.004
S[w/w%]	0.036	0.074	0.027	0.12	0.039	0.084	0.06	0.03	0.021	0.018
Impact Test (J)	6 (3–10)	71 (56–84)	18 (15–21)	6 (4–12)	7 (6–10)	9 (8–9)	12 (7–19)	-	14 (12–17)	8 (7–9)

**Table 4:** Results Corbones: number of cycles under fatigue dynamic test (10 Hz, R:0.1).

No. Cycles (200 MPa, 10 Hz, R: 0.1)			
Sample 1 Stringers:	820,006	Sample 1 Chords:	301,452
Sample 2 Stringers:	71,909	Sample 2 Chords:	984,618
Sample 3 Stringers:	517,416	Sample 3 Chords:	577,975
Sample 4 Stringers:	367,332	Sample 4 Chords:	794,206
Sample 5 Stringers:	271,047	Sample 5 Chords:	311,040

**Table 5:** Results (Corbones): Avg. growth per cycle, cycles per mm of crack growth.

Corbones-Stringers-	Corbones-Main Chords-
Avg. growth per cycle: 0.00000373 mm	Avg. growth per cycle: 1.3474 × 10^−6^ mm
Cycles per mm of growth: 268,356	Cycles per mm of growth: 742,127

**Table 6:** Results Bembézar: number of cycles under fatigue dynamic test (10 Hz, R:0.1).

No. Cycles (200 MPa, 10 Hz, R: 0.1)			
Sample 1 Stringers:	2,315,105	Sample 1 Chords:	153,871
Sample 2 Stringers:	192,268	Sample 2 Chords:	1,151,432
Sample 3 Stringers:	587,475	Sample 3 Chords:	1,046,938
Sample 4 Stringers:	445,612	Sample 4 Chords:	1,163,466
Sample 5 Stringers:	214,773	Sample 5 Chords:	1,244,549

**Table 7:** Results (Bembézar): Avg. growth per cycle, cycles per mm of crack growth.

Bembézar-Stringers-	Bembézar-Main Chords-
Avg. growth per cycle: 0.00000416 mm	Avg. growth per cycle: 0.000003 mm
Cycles per mm of growth: 268,356	Cycles per mm of growth: 330,037

**Table 8:** Summary results for the seven metal bridges.

	Puddle Iron	Steel (1905–1910)		Steel (1917–1920)			Steel (>1925)			
Metallic Bridge:	“Carballo”	“Caudal”	“La Peña”	“Colera”	“Bémbezar” Stringers	“Bémbezar” Chords	“Tea” Stringers	“Tea” Chords	“Corbones” Stringers	“Corbones” Chords
YS (0.2%) (MPa)	312.8 (297–324)	283.4 (272–289)	329.2 (321–342)	313.2 (271–351)	269.3 (261–277)	307 (300–314)	253.6 (242–266)	-	293 (289–299)	255 (254–356)
UTS (MPa)	367.4 (355–375)	381.2 (375–388)	418 (416–421)	379 (358–400)	332 (331–333)	367 (366–368)	405 (337–463)	-	409.3 (404–418)	382.5 (380–385)
Elongation (%)	9 (8–10)	35 (34–36)	32 (30–33)	35 (30–42)	39 (37–41)	32 (31–32)	30 (26–32)	-	34 (33–34)	35 (35)
C[w/w%]	0.016	0.068	0.095	0.049	0.029	0.054	0.0252	0.074	0.02	0.012
P[w/w%]	0.12	0.065	0.084	0.123	0.036	0.054	0.012	0.014	0.006	0.004
S[w/w%]	0.036	0.074	0.027	0.12	0.039	0.084	0.06	0.03	0.021	0.018
Impact Test (J)	6 (3–10)	71 (56–84)	18 (15–21)	6 (4–12)	7 (6–10)	9 (8–9)	12 (7–19)	-	14 (12–17)	8 (7–9)
Fatigue 150 MPa (Cycles/mm)	-	979,556	-	-	-	-	541,645	653,337	-	-
Fatigue 200 MPa (Cycles/mm)	628,393	34,934	157,747	-	268,356	74,227	60,807	57,881	330,037	240,790
Fatigue 250 MPa (Cycles/mm)	-	-	191,156	186,534	-	-	-	-	-	-

## Data Availability

Data available on request due to restrictions.
